# Effects of Hyperglycemia and Diabetes Mellitus on Coagulation and Hemostasis

**DOI:** 10.3390/jcm10112419

**Published:** 2021-05-29

**Authors:** Xiaoling Li, Nina C. Weber, Danny M. Cohn, Markus W. Hollmann, J. Hans DeVries, Jeroen Hermanides, Benedikt Preckel

**Affiliations:** 1 Department of Anesthesiology, Amsterdam UMC Location AMC, University of Amsterdam, 1105 AZ Amsterdam, The Netherlands; x.li1@amsterdamumc.nl (X.L.); n.c.hauck@amsterdamumc.nl (N.C.W.); m.w.hollmann@amsterdamumc.nl (M.W.H.); j.hermanides@amsterdamumc.nl (J.H.); 2 Department of Vascular Medicine, Amsterdam UMC Location AMC, University of Amsterdam, 1105 AZ Amsterdam, The Netherlands; d.m.cohn@amsterdamumc.nl; 3 Department of International Medicine, Amsterdam UMC location AMC, University of Amsterdam, 1105 AZ Amsterdam, The Netherlands; j.h.devries@amsterdamumc.nl

**Keywords:** metabolic disorder, platelets, coagulation factors, hypercoagulation, hypofibrinolysis

## Abstract

In patients with diabetes, metabolic disorders disturb the physiological balance of coagulation and fibrinolysis, leading to a prothrombotic state characterized by platelet hypersensitivity, coagulation disorders and hypofibrinolysis. Hyperglycemia and insulin resistance cause changes in platelet number and activation, as well as qualitative and/or quantitative modifications of coagulatory and fibrinolytic factors, resulting in the formation of fibrinolysis-resistant clots in patients with diabetes. Other coexisting factors like hypoglycemia, obesity and dyslipidemia also contribute to coagulation disorders in patients with diabetes. Management of the prothrombotic state includes antiplatelet and anticoagulation therapies for diabetes patients with either a history of cardiovascular disease or prone to a higher risk of thrombus generation, but current guidelines lack recommendations on the optimal antithrombotic treatment for these patients. Metabolic optimizations like glucose control, lipid-lowering, and weight loss also improve coagulation disorders of diabetes patients. Intriguing, glucose-lowering drugs, especially cardiovascular beneficial agents, such as glucagon-like peptide-1 receptor agonists and sodium glucose co-transporter inhibitors, have been shown to exert direct anticoagulation effects in patients with diabetes. This review focuses on the most recent progress in the development and management of diabetes related prothrombotic state.

## 1. Introduction

In 2017, the International Diabetes Federation estimated that 451 million adults are diagnosed with diabetes mellitus (DM) worldwide, and the number would increase to 693 million by 2045 [1]. Anesthesiologists are increasingly facing high-risk patients with significant comorbidities undergoing major surgery, including a significant risk for excessive bleeding, and hyperglycemia is associated with higher risk of perioperative complications and poorer outcomes after surgery [2,3]. The hemostatic function of platelets and coagulation factors sometimes makes it difficult to control the pro-thrombotic state since global inhibition of coagulation will impair hemostasis [4]. It is essential to understand the signalling partners and factors involved in platelet hyperresponsiveness or aberrant activation of the coagulation system. This will eventually allow for selective targeting of pro-thrombotic cascades while preserving hemostasis. Metabolic disorders influence coagulation and hemostasis, but the underlying mechanisms have yet to be clarified [5,6,7]. Besides, the choice and optimal dosage of antithrombotic agents for patients with DM are still unclear [8]. This review focuses on coagulation dysfunction and prothrombotic states in patients with DM.

## 2. Prothrombotic State Associated with Diabetes Mellitus

Coagulation and hemostasis involve interactions between tissue and coagulation factors as well as blood and endothelial cells, finally resulting in formation of fibrin clots stopping bleeding [4]. During this process, the fibrinolytic system decomposes generated clots to prohibit widespread thrombus formation and vascular occlusion [5].

**Figure 1:**
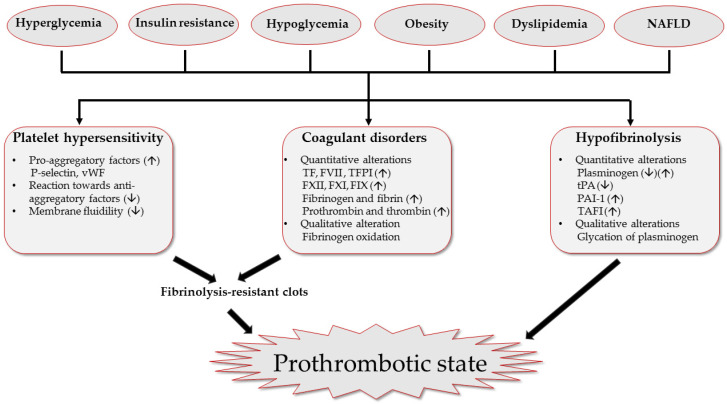
Modifications of coagulation and fibrinolysis system in DM. Diabetes disturbs the physiological balance between coagulation and fibrinolysis, leading to a prothrombotic state hallmarked with platelet hypersensitivity, coagulation factor disorders and hypofibrinolysis. Hyperglycemia and insulin resistance enhance number and aggregation of platelets through increasing von Willebrand factor (vWF) level and inhibiting anti-aggregatory efficiency of nitric oxide and prostaglandin I_2_. Hyperglycemia and insulin resistance upregulate level of pro-coagulation mediators like tissue factor (TF), coagulation factors (FVII, FXII, FXI and FIX) and thrombin. Diabetes also harasses fibrinolysis by decreasing tissue plasminogen activator (tPA) as well as increasing plasminogen activator inhibitor-1 (PAI-1) and thrombin activator fibrinolysis inhibitor (TAFI). Next to hyperglycemia and insulin resistance, co-existing metabolic disorders like hypoglycemia, obesity, dyslipidemia, and non-alcoholic fatty liver disease (NAFLD) also contribute to the pro-thrombotic state of patients with DM.

In patients having DM, metabolic disorders disturb these physiological mechanisms, leading to a prothrombotic state characterized by platelet hypersensitivity, coagulation factor disorders and hypofibrinolysis [9] (Figure 1). 

### 2.1. Platelet Hypersensitivity 

In physiological conditions, platelets circulate in the blood for five to seven days and constantly undergo a lifecycle from megakaryocyte separation to phagocytosis by macrophages, to maintain a normal platelet count of 150.000–450.000 per microliter. After vascular injury, platelets are activated to aggregate, forming an occlusive thrombus and stop bleeding [10]. Both increased platelet number and enhanced aggregation capacity, the latter referred to as platelet hypersensitivity, will contribute to a pro-thrombotic state [11]. In patients with DM, over-activation of platelets (mainly attributed to hyperglycemia and insulin resistance) plays a crucial role for pro-thrombotic events [12]. 

#### 2.1.1. Hyperglycemia

Among patients with type 2 diabetes mellitus (T2DM), mean platelet counts are up to 10% higher in patients with chronic hyperglycemia (glycated hemoglobin, HbA1c > 8%) as compared to euglycemia. Besides, increased mean platelet volume (MPV) is observed in patients with higher HbA1c and fasting blood glucose (FBG), suggesting that hyperglycemia also enhances platelet activity [13]. Similarly, positive correlations between higher blood glucose and increased platelet counts are also found in patients with type 1 diabetes mellitus (T1DM) [14,15]. Even in patients in a pre-diabetic state, MPV is slightly increased compared to patients with normal glucose metabolism (10.49 ± 0.96 fL vs. 10.04 ± 1.01 fL) [16]. A potential underlying mechanism for platelet hyperactivity in hyperglycemia could be an upregulated expression of pro-aggregatory factors like P-selectin, thromboxane A_2_ and von Willebrand factor (vWF) antigen, amplifying the aggregation and adhesion of platelets [17]. 

In addition, high blood glucose deteriorates the physiologic reaction of platelets on anti-aggregatory effects of nitric oxide (NO), prostaglandin I_2_ (PGI_2_) and insulin by interfering with downstream signalling pathways [18]. Both acute and chronic hyperglycemia upregulate the expression of adhesion molecules on platelet surface (e.g., CD31, CD49b and CD63), an effect that is reversible after optimizing glucose control [19]. Chronic hyperglycemia increases expression of protease-activated receptor 4 in patients with DM, in turn promoting the release of activated platelet-derived microparticles (PMPs) via the Ca^2+^-calpain pathway. Released PMPs then trigger the secretion of interleukin-6, a pro-thrombotic and pro-inflammatory mediator in diabetes [20]. Through glycation of membrane proteins, hyperglycemia also decreases the membrane fluidity of platelets and results in increased intracellular calcium influx, directly promoting platelet activation and aggregation [21]. Hyperglycemia also impairs endothelial function by inducing inflammation and oxidative stress, thereby inhibiting synthesis and release of PGI_2_ and NO, finally further promoting platelet aggregation [22].

#### 2.1.2. Insulin Resistance 

Patients with T2DM had larger MPV and increased platelet generation compared to patients having T1DM; MPV correlated with HbA1c only in patients with T1DM [17]. Given that T1DM is characterized by insulin deficiency and T2DM is hallmarked with insulin resistance, these data might indicate a potential role of insulin sensitivity and resistance in coagulation dysfunction [23]. Another study showed a positive correlation between MPV and homeostasis model assessment insulin resistance index [24]. A possible underlying mechanism is a modified insulin signalling in platelets associated with insulin resistance: in healthy individuals, insulin binds with insulin receptors on platelet surfaces, leading to activation of downstream pathways, e.g., the tyrosine phosphorylation pathway and the inhibitory G-protein pathway [25]. The latter results in higher cyclic adenosine monophosphate (cAMP) generation and lower intracellular calcium inside platelets, thereby inhibiting their aggregation [18]. 

However, in patients with insulin resistance, insulin failed to increase cAMP levels within platelets, thus impairing its anti-aggregational effects [26]. Insulin resistance also reduces the sensitivity of platelets towards the anti-aggregatory effects of NO and PGI_2_, which in turn alters calcium influxes and promotes platelet aggregation [27]. Intriguingly, recent studies linked insulin resistance with an altered composition of gut microbiomes [28], and the latter contributes to the enhanced thrombosis risk via generation of platelet stimulus metabolites [29]. For instance, trimethylamine N-oxide might directly increase the aggregation and adhesion capacity of platelet [30], and phenylacetylglycine facilitates platelet responsiveness and enhances thrombosis tendency [31]. Antidiabetic drugs may be able to change the microbiome structure and the resulting plasma metabolome. Thus, pleiotropic effects observed by, e.g., metformin, could at least in part be mediated through microbiota and their released metabolites and that may include antithrombotic effects of the drug. This is an active area of research and must show in the near future whether antidiabetics can play a substantial role in these pathomechanisms [32].

### 2.2. Quantitative and Qualitative Alterations of Coagulation Factors

Coagulants (also known as coagulation factors) are a series of proteins involved in the coagulation process, working together with platelets to form a firm clot and stop bleeding [33]. Both, quantitative and qualitative alterations of coagulation and anticoagulation factors were observed in patients with DM [34], contributing to formation of lysis-resistant clots. 

The extrinsic coagulation or Tissue Factor (TF) pathway is initialized with activation of tissue factor/Factor VIIa (TF/FVIIa) complexes and plays an essential role in thrombus generation [35]. In T2DM, hyperglycemia and insulin resistance exert synergistic effects on the TF pathway, leading to increased pro-coagulatory activity and FVIIa consumption [36]. Several mechanisms are involved: hyperglycemia and hyperinsulinemia both directly promote the TF transcription in monocytes [37]. Additionally, patients with diabetes are prone to constant inflammation, and one symptom is increased inflammatory biomarker levels (e.g., interleukins) in blood [38]. The pathological process directly upregulates TF expression in endothelial and vascular smooth muscle cells, contributing to the hypercoagulation state in DM [39]. Generation of advanced glycation end-products, glycated lipids or proteins formed during hyperglycemia [40], as well as reactive oxygen species enhance TF production through activation of the nuclear factor (NF)-κB inflammatory pathway [41]. Notably, recent studies highlight the role of micro-RNA (miR) in TF expression and diabetes-related coagulation dysfunction. For instance, miR-126, miR-19a and miR-181b are negatively associated with both TF protein and TF-mediated thrombogenicity in patients with diabetes. Further investigations showed that miR-126 and miR-19a both inhibit the TF expression in endothelial cells, and miR-181b reduced the generation of TF within monocytes [42,43,44]. These findings might explain in part the enhanced vascular TF activity in poorly controlled type 2 diabetes. 

The intrinsic coagulation pathway involves sequential activations of FXII, FXI and FIX, and recent studies have reported dysfunctions of intrinsic coagulation associated with hyperglycemia and hyperinsulinemia in DM [45,46]. In the Netherlands’ Epidemiology of Obesity study [46], increases in FVIII (5.33%, 95%CI: 4.00–6.65), FIX (6.19%, 95%CI: 5.15–7.23) and FXI (2.11%, 95%CI: 1.20–3.02) were observed per mmol/L increase in fasting plasma glucose, and these associations remained significant even after adjusting for confounding factors such as age, sex and body mass index (BMI). Increased synthesis of FXII, FXI and FIX in hepatocytes along with a shorter activated partial thromboplastin time (APTT) were observed in patients with impaired insulin sensitivity, probably mediated by a low-grade inflammatory reaction caused by insulin resistance [47].

Conversion of fibrinogen to fibrin is the last critical step in extrinsic and intrinsic coagulation pathways, and higher circulating fibrinogen levels are observed in T1DM and T2DM patients, resulting in a more compacted clot structure along with increased resistance to fibrinolysis [48]. Hyperfibrinogenemia in DM can be explained by several factors. Hyperglycemia and insulin resistance enhance hepatic fibrinogen synthesis, and production of fibrinogen is further increased in obesity, dyslipidemia and non-alcoholic fatty acid diseases, all of which are common comorbidities in hyperglycemic patients [49]. In addition, low-grade inflammation in DM directly stimulates hepatocytes to synthesize more fibrinogen [50].

Modified quality of fibrinogen in DM has recently been demonstrated: an increased glucose level amplifies glycation of fibrinogen and disturbs the fibrinolytic process, and these alterations could be attenuated by tight glucose control [51]. Enhanced oxidative stress and sustained inflammatory reactions in patients having DM also alter the structure of fibrinogen and fibrin, thereby leading to fibrinolysis-resistant clots [52]. 

Thrombin is derived from prothrombin and is an essential factor that transforms fibrinogen into fibrin. In patients with DM, increased thrombin levels lead to enhanced fibrin generation and clot density, thereby contributing to the pro-thrombotic state [53]. Both hyperglycemia and hyperinsulinemia stimulate prothrombin synthesis in the liver. Recent studies reported increased thrombin levels in obesity and hyperlipidemia, comorbidities that can therefore aggravate the hypercoagulable state in patients with DM [54,55]. 

### 2.3. Hypofibrinolysis

The hypofibrinolytic state in DM is partly attributed to the formation of fibrinolysis-resistant clots; however, alterations of the fibrinolytic system have also been observed among individuals with diabetes [56].

Plasminogen is the precursor of plasmin. During coagulation, generated fibrin binds with tissue plasminogen activator (tPA), the key catalysator of plasmin generation, which then initiates fibrinolysis and restrains excessive thrombus formation [57]. In diabetic patients with DM, tPA is negatively correlated with HbA1c (lower levels of tPA with higher levels of HbA1c); increased glucose levels inhibit tPA’s pro-fibrinolytic activity, leading to reduced levels of plasmin in blood with high glucose values [56]. Hyperglycemia enhances the glycation of plasminogen and thereby inhibits the generation of plasmin, an effect that is reversible after tight glucose control [58]. The impact of plasminogen on coagulation disorders in DM is further aggravated as plasminogen is also a pro-inflammatory factor, thereby promoting insulin resistance and exacerbating a pro-thrombotic state [59]. 

Plasminogen activator inhibitor-1 (PAI-1) and thrombin-activator fibrinolysis inhibitor (TAFI) are two crucial inhibitory factors of the fibrinolytic system. PAI-1 forms complexes with tPA to harass its catalytic capacity, while TAFI prevents plasminogen from binding to fibrin and terminates the conversion of plasminogen to plasmin [57]. Hyperglycemia and insulin resistance lead to elevation of these two anti-fibrinolytic factors, resulting in reduced fibrinolytic factors [60]. The alterations of PAI-1 and TAFI could be reversed by euglycemia, emphasizing the importance of glucose control in alleviating the fibrinolytic dysfunction in DM [56]

## 3. Prothrombotic Effects of Coexisting Metabolic Disorders

DM is a cluster of metabolic disorders, and next to hyperglycemia and insulin resistance, co-existing changes like hypoglycemia, obesity and dyslipidemia also contribute to the pro-thrombotic state of patients with DM.

### 3.1. Hypoglycemia

Hypoglycemia is a crucial acute complication of DM treatment and it significantly increases cardiovascular risk and mortality [61]. Hypoglycemia is also associated with increased thrombus formation based on platelet activation, quantitative and qualitative alterations of coagulants, as well as impaired fibrinolysis [62,63]. In T2DM patients, platelet function increased with decreasing blood glucose and increasing epinephrine, suggesting that hypoglycemia induces platelet activation by sympathetic stimulation [64]. Hypoglycemia also promotes coagulant and fibrinolytic dysfunction by inducing pro-inflammatory reactions and impairing endothelial function in patients with DM [65]. Increased thrombogenicity after hypoglycemia lasted for up to seven days [66], indicating that hypoglycemia might exert short- to medium-term harmful effects on coagulation function. 

### 3.2. Obesity

Overweight and obesity, according to World Health Organization guidelines defined as BMI ≥ 25 kg/m^2^ and 30 kg/m^2^, respectively, are common comorbidities of two-thirds of patients with DM [67]. Obesity is considered a noticeable pro-thrombotic factor: increased thrombogenicity with higher BMI, and a hazard ratio for thrombosis of 3.4 (95% CI: 2.6–4.6) was observed in severe obese subjects compared with those of normal-weight [68]. Underlying mechanisms could be the increased number and size of adipocytes in obese individuals, accompanied by enhanced secretion of TF and PAI-1, finally leading to hyper-coagulation and hypo-fibrinolysis [69,70]. Additionally, increased body weight causes physical inactivity and slows down blood flow with stasis, resembling a pro-thrombotic factor by itself [68]. 

### 3.3. Dyslipidemia

Dyslipidemia is common among patients with T2DM and is associated with hypercoagulation [71]. Individuals with high triglyceride and total cholesterol levels showed higher fibrinogen levels and shortened APTT, suggesting an enhanced endogenous potential for thrombin generation [72]. Coagulation dysfunction of patients with DM was partially alleviated by lipid-lowering agents [53]. A potential underlying mechanism could be increased blood lipids, which directly impair function of hepatic cells (e.g., the origins of multiple coagulation factors) and in turn disturb coagulation [73]. Oxidized low density cholesterol also activates scavenger receptors on monocytes, resulting in inflammatory pathway activation and overproduction of oxidants and coagulation factors [74]. 

### 3.4. Nonachoholic Fatty Liver Disease (NAFLD)

Over 50% of patients with T2DM suffer from NAFLD, which may contribute to coagulation disorders and also a pro-thrombotic state [75]. Given that hepatic cells are the major producer for multiple coagulants (e.g., VIII, XI, XII), accumulation of liver fat might correlate with changes in coagulation factor production and prolonged APTT in patients with NAFLD [76,77]. Contrarily, another study showed that insulin resistance and adipose tissue inflammation, rather than liver fat content, enhanced the expression and activity of coagulant factors [47].

## 4. Management of Pro-Thrombotic State in DM

Due to the prothrombotic state and enhanced cardiovascular risk in diabetic patients, the necessity of antiplatelet and anticoagulation therapy is well acknowledged, but the optimal treatment strategies remain controversial [78,79]. In addition, optimized glucose-lipid control and weight loss further alleviate the pro-thrombotic state in DM [80]. 

### 4.1. Antiplatelet Medications

Aspirin is the most widely used antiplatelet agent also in patients with DM [79]. It binds irreversibly to cyclooxygenase-1 (COX-1) on platelets and prevents the generation of prostaglandin H_2_ from arachidonic acid, thus inhibiting thromboxane A_2_ formation and platelet activation [81]. Aspirin (75–162 mg per day) is recommended as the first choice for secondary prevention in patients with a history of cardiovascular disease (grade A) [82], but the most recent guidelines of the American College of Cardiology and the American Heart Association do not specifically recommend aspirin as a primary prevention strategy in diabetes patients [82]. A meta-analysis showed that aspirin reduced the first cardiovascular event by around 12% in patients without a history of cardiovascular disease, which was less effective compared to its effects in secondary prevention (22% reduction of cardiovascular events) [83]. One possible explanation might be that the absolute incidence of a very first cardiovascular event is low in patients without previous cardiovascular disease, thereby impairing the efficiency of aspirin in the primary prevention of cardiovascular events [84]. P2Y_12_ inhibitors like clopidogrel are commonly combined with aspirin for dual antiplatelet therapy (DAPT), which is recommended (grade A) also for patients suffering from acute coronary syndrome [82]. Clopidogrel is also considered as a substitute in patients with contraindications for aspirin [85]. 

Population-based clinical trials have been carried out to explore the efficacy and safety of other antiplatelet drugs in patients with DM. Cilostazol, a phosphodiesterase III inhibitor enhancing cAMP generation and preventing platelet aggregation, decreased stent thrombosis after stent-implantation in patients having DM when combined with DAPT [86]. Compared to aspirin, the thromboxane synthase inhibitor picotamide reduced non-fatal vascular complications in T2DM patients with peripheral vascular disease [87]. However, neither cilostazol nor picotamide were superior to aspirin in preventing overall mortality [87,88], thus aspirin is still the first-choice antiplatelet treatment in patients with a history of cardiovascular disease. 

### 4.2. Anticoagulation Medications

Currently, anticoagulants such as thrombin-inhibitors or anti-Xa agents are only recommended in patients with diabetes in case they experience complications, e.g., thrombosis or high risk for thrombotic tendency (e.g., atrial fibrillation) [82]. Warfarin non-specifically inhibits the synthesis of several clotting factors by inhibiting the vitamin K epoxide reductase complexes [89]. Direct oral anticoagulants (DOACs) target thrombin or factor Xa and exert specific anticoagulation activities [90]. Several studies have compared the effectiveness and safety of DOACs and warfarin in patients at a high risk for thrombotic diseases. In individuals with and without DM, Rivaroxaban, a specific inhibitor of FXa, is associated with a lower risk of systemic embolism and major bleeding than warfarin [91,92]. Low-dose rivaroxaban also reduced platelet activation and inflammation via the inhibition of the binding of FXa towards protease-activated receptors on platelet surfaces [93], possibly ameliorating the pro-thrombotic state in patients with diabetes with no indication for a full anticoagulant dose. Patients on apixaban, another FXa inhibitor, experienced 30% less incidents of hemorrhages than those exposed to warfarin [94]. The thrombin inhibitor dabigatran also leads to more potent reduction in embolic events in individuals with DM than warfarin, without increasing the risk of hemorrhage [95]. These studies suggest that DOACs might serve as more potent and safer alternatives to warfarin for anticoagulation therapy in patients with DM.

### 4.3. Metabolism Optimization

#### 4.3.1. Glucose Control

Several glucose-lowering agents exert glucose-independent anticoagulation effects in patients with DM. Alleviation of coagulation dysfunction is mediated by improvement of insulin resistance, endothelial function, inflammatory reaction and oxidative stress. [96,97,98].

For instance, metformin, the most widely prescribed anti-diabetes drug, lowers the activity of coagulation factors and platelets and prevents the formation of fibrinolysis-resistant clots [99,100]. The attenuation of coagulation function was explained by an enhancement of insulin sensitivity and a normalization of endothelial function for metformin treatment [96]. A recent study revealed that metformin directly lowered expression and activity of TF in patients with chronic hyperglycemia and poorly controlled glucose, which was mediated by suppression on endothelial inflammation [97]. Thiazolidinediones have also been proven to attenuate coagulation dysfunction by reducing fibrinogen and PAI-1 levels, thereby modulating the balance between clot generation and fibrinolysis [101]. Rosiglitazone inhibited platelet aggregation activity in a dose-dependent manner, mediated by enhanced insulin sensitivity and decreased inflammatory reaction and oxidative stress [98]. Considering sulphonylureas, glibenclamide dose-dependently inhibited the expression of TF, producing a potential anticoagulation effect of sulphonylureas [102]. Insulin inhibits platelet aggregation by inducing cAMP generation and reducing intracellular calcium levels [18]. Contrarily, in patients with T2DM, insulin treatment was associated with higher fibrinogen and PAI-1 levels due to insulin resistance [103]. 

The anticoagulation potential of glucagon-like peptide-1 receptor agonists (GLP-1 RAs), a group of glucose-lowering agents with cardiovascular protective effects [104], has been demonstrated; after binding with GLP-1 receptors on platelets, liraglutide increased the sensitivity of platelets towards NO, thereby inhibiting aggregation activity of platelets [105]. Apart from glucose control, GLP-1 RA also exerts anticoagulation effects by inhibiting inflammation and promoting NO synthesis of endothelial cells [106]. 

Sodium glucose co-transporter 2 inhibitors (SGLT-2i’s) are also glucose-lowering drugs with beneficial cardiovascular effects. These drugs directly alleviate DM-related endothelial dysfunction, one of the major modulators of coagulation and fibrinolysis [107,108]. A recent study in human endothelial cells demonstrated that empagliflozin and dapagliflozin restored NO bioavailability [109], which is an anticoagulation factor inhibiting platelet aggregation [22]. More recently, empagliflozin has also been shown to decrease the plasma concentration of PAI-1 in patients with T2DM (by 25%), thereby improving fibrinolysis function [110]. Intriguingly, two clinical trials (NCT04342819 and NCT04400760) are now performed, recruiting patients with DM to figure out the influence of empagliflozin and dapagliflozin on platelet functions; these studies will present more insight into the anticoagulation potential of SGLT-2i’s. The direct anticoagulation effects of mentioned glucose-lowering agents have been summarized in Table 1. 

#### 4.3.2. Weight Loss

Diet, exercise and bariatric surgery are common weight loss therapies [111], and their antithrombotic effects have also been explored [112,113,114]. Gastric bypass surgery reduced thrombin generation activity by one-third after six months, which is partially mediated by significant weight loss (27.4 ± 0.7 kg) and improved glucose-lipid metabolism benefits [112]. The combination of surgery with supervised physical training further improved coagulation and metabolic benefits [112]. Mild weight reduction (within 5 kg) induced by leisure exercise and dietary modulation failed to reduce thrombogenic markers in overweight/obese subjects [113,114], suggesting that the influence of weight loss on coagulation might be determined by the extent of weight loss. A recent study showed that lifestyle intervention led to reduced activities of multiple coagulation factors (II, VII, VIII, IX, XI and XII) and decreased levels of PAI-1 and vWF. These beneficial changes in the coagulation system were not only mediated through weight loss, but also via the ameliorated insulin resistance and limited subclinical inflammation [115].

#### 4.3.3. Lipid-Lowering Therapy

The anticoagulation functions of lipid-lowering agents have been described: statin-lowering therapy led to reductions of thrombotic risk and platelet activity in patients with DM [53,116]. For instance, fibrates and ezetimibe alleviated hypercoagulability in patients with impaired glucose metabolism, probably mediated by lipid-lowering and anti-inflammatory effects[117]. Lipid-lowering agents also directly reduced the expression levels of several pro-coagulation factors, e.g., TF and P-selectin, thereby inhibiting thrombin generation [118]. Moreover, proprotein convertase subtilisin/kexin type-2 (PCSK9) inhibitors, potent agents against hypercholesterolemia, might enhance the expression of hepatic low-density lipoprotein receptors and accelerate FVIII degradation within the liver, thereby lowering circulating FVIII levels [119]. However, at present, there is no direct proof for the anticoagulation effect of PCSK9 inhibitors and the decreased FVIII in patients with DM.

## 5. Conclusions

In summary, patients with T1DM and T2DM are prone to thrombotic events based on a series of disorders, including platelet hypersensitivity, coagulation factor modifications and hypofibrinolysis. Studies on the altered coagulation in DM suggest that hyperglycemia, insulin resistance and other comorbidities contribute to the hypercoagulable state (Figure 1). Thus, management of the enhanced thrombogenicity in DM requires comprehensive treatments of existing prothrombotic factors along with antithrombotic therapy, but current guidelines lack recommendations on the optimal antithrombotic medications in patients with DM. Results from clinical studies strongly support the beneficial effects of glucose control, weight loss and lipid-lowering on coagulation dysfunctions, and glucose-lowering drugs, especially GLP-1 RAs and SGLT-2i’s, tend to ameliorate diabetes related hypercoagulation (Table 1). 

## Figures and Tables

**Table 1:** Anticoagulation effects of glucose-lowering agents.

Agent	Alterations in Coagulation-Fibrinolysis System
Metformin	tPA ↓[83]; platelet aggregation↓[84]; TF ↓[97]
Thiazolidinediones	fibrinogen↓; PAI-1↓[101] ; platelet aggregation↓[98]
Sulphonylureas	TF↓[102]
Insulin	platelet aggregation↓[18]; fibrinogen↑; PAI-1↑[103]
GLP-1RA	platelet aggregation↓[105]; NO↑[106]
SGLT-2i’s	NO↑[109]; PAI-1↓[110]

GLP-1RA: glucagon-like peptide-1 receptor agonists; SGLT-2i’s: sodium glucose co-transporter 2 inhibitors; tPA: tissue plasminogen activator; TF: tissue factor; PAI-1: plasminogen activator inhibitor-1; NO: nitric oxide.
